# Supplementary material for: Infant Exposure to Antituberculosis Drugs via Breast Milk and Assessment of Potential Adverse Effects in Breastfed Infants: Critical Review of Data
Source: Pharmaceutics. 2023 Apr 13;15(4):1228. doi: 10.3390/pharmaceutics15041228 (PMC10143885; doi:10.3390/pharmaceutics15041228)
Supplement: Supplementary file 1 [file pharmaceutics-15-01228-s001.zip › pharmaceutics-2284381-supplementary.pdf]

Supplementary data

Supplementary Table S1. Search string for literature collection in PubMed.

	**Database**<br><br>**PubMed**
**Keywords for the substances**	Isoniazid<br>Rifampicin<br>Ethambutol<br>Pyrazinamide<br>Streptomycin<br>Levofloxacin<br>Bedaquiline<br>Linezolid<br>Clofazimine<br>Cycloserine<br>Terizidone<br>Pretomanid/PA824
**Keywords for kinetics, plasma concentration, blood concentration**	pharmacokinetics [MeSH term]; plasma concentration [Title/Abstract] OR plasma concentrations Title/Abstract]
**Keywords for milk concentration**	human milk concentration [MeSH term];
**Keywords for infant plasma concentration**	infant plasma concentration [MeSH term];

Supplementary Table S2. Checklist for quality assessment of clinical pharmacokinetic studies according to Gafar et al. (Gafar et al., 2022)

Appraising Background			
1.	Was a clear description of the objectives of the study provided?	Yes/No/NA	1/0/0
2.	Was a clear and comprehensive rationale provided to support the purpose of the study?	Yes/No/NA	1/0/0
Appraising Study Design and Experimental Methods			
3.	Was the chosen study design appropriately selected and justified?	Yes/No/NA	1/0/0
4.	[Slightly modified from the original version] Was the description of at least the drug dose (in mg or mg/kg of body weight) and dosing interval (single-dose, daily, or intermittent [trice weekly] dose, etc.), with addition of drug administration (taken whole by mouth, crushed/dispersed and taken via syringe/nasogastric tube, etc.) justified for the intended study?	Yes/No/NA	1/0/0
5.	Were the outcome measures endpoints of the study appropriate to address the objectives of the study?	Yes/No/NA	1/0/0
7.	Were the exclusion criteria of participants included and appropriate for the intended outcomes of the study?	Yes/No/NA	1/0/0
8.	Were the relevant baseline characteristics of the participants adequately described?	Yes/No/NA	1/0/0
9.	Were plausible interacting covariates described *a priori* or in post hoc evaluation?	Yes/No/NA	1/0/0
10.	Was the description of the used biological sample analytical methods or citations of prior validation studies provided in the publication or affiliated appendix?	Yes/No/NA	1/0/0
11.	Was the method of data sampling of analytics appropriate for the study?	Yes/No/NA	1/0/0
12.	Was a clear description of the sampling site provided and justified?	Yes/No/NA	1/0/0
13.	[Slightly modified from the original version] Was the number of samples taken within the sampling period appropriate for the assessment of total plasma exposure (i.e., area under the concentration-time curve from 0-24 h post-dose [AUC0-24]), including assessment of AUC0-24 using non-	Yes/No/NA	1/0/0

	compartmental pharmacokinetic analysis or population pharmacokinetic modelling?		
14.	Were sample storage conditions appropriate and described in a manner that could be accurately replicated?	Yes/No/NA	1/0/0
15.	If applicable, was there a clear description of the pharmacokinetic model, its development, validation and justification for use.	Yes/No/NA	1/0/0
16.	If applicable, was the described population pharmacokinetic approach validation method appropriate for the analysis?	Yes/No/NA	1/0/0
17.	Were the essential pharmacokinetic parameters required to make the results applicable in clinical settings included?	Yes/No/NA	1/0/0
18.	Were the pharmacokinetic equations used to calculate the patient's pharmacokinetic parameters presented or cited within the article?	Yes/No/NA	1/0/0
Appraising Applied Statistics			
19	Were the chosen statistical tests and software to perform the statistical analysis appropriate to achieve the study objectives?	Yes/No/NA	1/0/0
Appraising Results			
20	Were all patients enrolled in the study accounted for?	Yes/No/NA	1/0/0
21	In the event of missing data or outliers, was the process for analysis justified and appropriate?	Yes/No/NA	1/0/0
22	Were appropriate summary statistics to describe centrality and variance used to present the pharmacokinetic results?	Yes/No/NA	1/0/0

Supplementary Table S3. Relevant parameters used in the calculation of milk/plasma ratio for pretomanid.

Parameter	Value	Reference
**pH plasma**	7.4	(Atherton, 2009)
**pH milk**	6.60 ± 0.28	(Filatava et al., 2023)
**Crematocrit**	9.53 ±3.98	(Meier et al., 2006)
**Fraction of fat in milk**	0.038-0.039	(Bobiński and Bobińska, 2022)
**Log $P_{O/W}$**	2.42	Assessment Report, 25 June 2020<br>EMA/200048/2020<br>Committee for Medicinal Products for Human Use (CHMP)
**Molecular weight**	359.26	PubChem
**pKa**	7.0	PubChem
**Fu plasma**	13.6	PubChem

Equations for calculation of M:P ratio according to Abduljalil et al. (Abduljalil et al., 2021)

$$f_p^{un} = \frac{1}{1 + 10^{(pKa\, -\, \mathrm{pH_{Plasma}})}}$$

$$f_{mk}^{un} = \frac{1}{1 + 10^{(pKa\, -\, \mathrm{pH_{Milk}})}}$$

$$\mathrm{fu_{skimmed}} = \frac{\mathrm{fu}_p{}^{0.448}}{0.000694^{0.448} + \mathrm{fu}_p{}^{0.448}}$$

$$\mathrm{fu}_{mk} = \frac{1}{\frac{fw}{\mathrm{fu_{skimmed}}} + f_{\mathrm{fat}} \cdot \mathrm{Log}\, P_{\mathrm{milk}}}$$

$$\frac{S}{W} = \frac{1}{1 + \mathrm{Crt}\ \cdot (\mathrm{fu}_{mk} \cdot \mathrm{Log}\, P_{o:w} - 1)}$$

$$\frac{M}{P} = \frac{\mathrm{fu}_p \cdot f_p^{un}}{f_{mk}^{un}} \cdot \frac{1}{f\mathrm{u}_{mk} \cdot \left(\frac{S}{W}\right)}$$

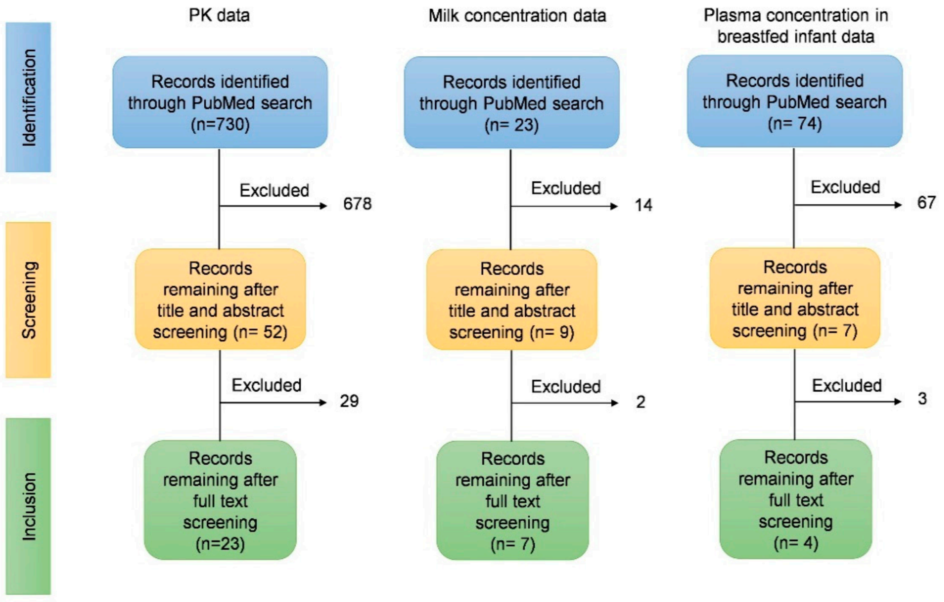

Supplementary Figure S1. Flowchart outlining the cumulative number of retrieved articles at each stage of the literature review for the pharmacokinetics, milk concentrations and breastfed infant's plasma concentration data of antituberculosis drugs.

ABDULJALIL, K., PANSARI, A., NING, J. & JAMEI, M. 2021. Prediction of drug concentrations in milk during breastfeeding, integrating predictive algorithms within a physiologically-based pharmacokinetic model. *CPT Pharmacometrics Syst Pharmacol,* 10**,** 878-889.

ATHERTON, J. C. 2009. Acid–base balance: maintenance of plasma pH. *Anaesthesia & Intensive Care Medicine,* 10**,** 557-561.

BOBIŃSKI, R. & BOBIŃSKA, J. 2022. Fatty acids of human milk - a review. *Int J Vitam Nutr Res,* 92**,** 280-291.

FILATAVA, E. J., SHELLY, C. E., OVERTON, N. E., GREGAS, M., GLYNN, R. & GREGORY, K. E. 2023. Human milk pH is associated with fortification, postpartum day, and maternal dietary intake in preterm mother-infant dyads. *J Perinatol,* 43**,** 60-67.

GAFAR, F., WASMANN, R. E., MCILLERON, H. M., AARNOUTSE, R. E., SCHAAF, H. S., MARAIS, B. J., AGARWAL, D., ANTWI, S., BANG, N. D., BEKKER, A., BELL, D. J., CHABALA, C., CHOO, L., DAVIES, G. R., DAY, J. N., DAYAL, R., DENTI, P., DONALD, P. R., ENGIDAWORK, E., GARCIA-PRATS, A. J., GIBB, D., GRAHAM, S. M., HESSELING, A. C., HEYSELL, S. K., IDRIS, M. I., KABRA, S. K., KINIKAR, A., HEMANTH KUMAR, A. K., KWARA, A., LODHA, R., MAGIS-ESCURRA, C., MARTINEZ, N., MATHEW, B. S., MAVE, V., MDUMA, E., MLOTHA-MITOLE, R., MPAGAMA, S. G., MUKHERJEE, A., NATAPRAWIRA, H. M., PELOQUIN, C. A., POUPLIN, T., RAMACHANDRAN, G., RANJALKAR, J., ROY, V., RUSLAMI, R., SHAH, I., SINGH, Y., STURKENBOOM, M. G. G., SVENSSON, E. M., SWAMINATHAN, S., THATTE, U., THEE, S., THOMAS, T. A., TIKISO, T., TOUW, D. J., TURKOVA, A., VELPANDIAN, T., VERHAGEN, L. M., WINCKLER, J. L., YANG,

H., YUNIVITA, V., TAXIS, K., STEVENS, J. & ALFFENAAR, J. C. 2022. Global estimates and determinants of antituberculosis drug pharmacokinetics in children and adolescents: a systematic review and individual patient data meta-analysis. *Eur Respir J*.

MEIER, P. P., ENGSTROM, J. L., ZULEGER, J. L., MOTYKOWSKI, J. E., VASAN, U., MEIER, W. A., HARTMANN, P. E. & WILLIAMS, T. M. 2006. Accuracy of a user-friendly centrifuge for measuring creamatocrits on mothers' milk in the clinical setting. *Breastfeed Med,* 1**,** 79-87.
